# Cardiovascular Events and Preoperative Beta-Blocker Use in Non-Cardiac Surgery: A Prospective Holter-Based Analysis

**DOI:** 10.3390/medicina61071300

**Published:** 2025-07-18

**Authors:** Alexandru Cosmin Palcău, Liviu Ionuț Șerbanoiu, Livia Florentina Păduraru, Alexandra Bolocan, Florentina Mușat, Daniel Ion, Dan Nicolae Păduraru, Bogdan Socea, Adriana Mihaela Ilieșiu

**Affiliations:** 1 Faculty of Medicine, Carol Davila University of Medicine and Pharmacy, 050474 Bucharest, Romania; livia.paduraru@umfcd.ro (L.F.P.); alexandra.bolocan@umfcd.ro (A.B.); florentina.musat@drd.umfcd.ro (F.M.); ion.daniel@umfcd.ro (D.I.); dan.paduraru@umfcd.ro (D.N.P.); bogdan.socea@umfcd.ro (B.S.); iliesiu.adriana@umfcd.ro (A.M.I.); 2 General Surgery Department, University Emergency Hospital of Bucharest, 050098 Bucharest, Romania; 3 Department of Cardiology, Emergency Hospital Bagdasar-Arseni, 050474 Bucharest, Romania; 4 Department of Cardiology, Elias Emergency University Hospital, 011461 Bucharest, Romania; 5 Department of General Surgery, Sf. Pantelimon Emergency Clinical Hospital, 021659 Bucharest, Romania; 6 Department of Cardiology, “Prof. Dr. Theodor Burghele” Clinical Hospital, 050659 Bucharest, Romania

**Keywords:** beta-blockers, non-cardiac surgery, atrial fibrillation, bradycardia, ventricular tachycardia, extrasystoles, hypotension, perioperative cardiac management

## Abstract

*Background and Objectives*: The perioperative use of beta-blockers remains controversial due to conflicting evidence of their risks and benefits. The aim of this study was to evaluate the association between chronic beta-blocker (bb) therapy and perioperative cardiac events in non-cardiac surgeries using 24 h continuous Holter monitoring. *Materials and Methods*: A prospective observational study was conducted on patients undergoing elective or emergency non-cardiac surgery at a Romanian tertiary care hospital. The patients were divided into two groups: G1 (not receiving Bb) and G2 (on chronic Bb). The incidences of perioperative cardiac events, such as severe bradycardia (<40 b/min), new-onset atrial fibrillation (AF), extrasystolic arrhythmia (Ex), and sustained ventricular tachycardia (sVT) and arterial hypotension, were compared between the two groups using clinical, electrocardiography (ECG), and Holter ECG data. Beta-blocker indications, complications, and outcomes were analyzed using chi-squared tests and logistic regression. *Results*: A total of 100 consecutive patients (63% men, mean age of 53.7 years) were enrolled in the study. G2 included 30% (*n* = 30) of patients on chronic beta-blocker therapy. The indications included atrial fibrillation (46.7%, *n* = 14), arterial hypertension (36.7%, *n* = 11), extrasystolic arrhythmias (10%, *n* = 3), and chronic coronary syndrome (6.6%, *n* = 2). Beta-blocker use was significantly associated with severe bradycardia (*n* = 6; *p* < 0.001) in G2, whereas one patient in G1 had bradycardia, and 15 and 1 patients had hypotension (*p* < 0.001) in G1 and G2, respectively. The bradycardia and arterial hypotension cases were promptly treated and did not influence the patients’ prognoses. The 14 patients with AF in G2 had a 15-fold higher odds of requiring beta-blockers (*p* < 0.001, odds ratio (OR) = 15.145). No significant associations were found between beta-blocker use and the surgery duration (*p* = 0.155) or sustained ventricular tachycardia (*p* = 0.857). Ten patients developed paroxysmal postoperative atrial fibrillation (AF), which was related to longer surgery durations (165 (150–180) vs. 120 (90–150) minutes; *p* = 0.002) and postoperative anemia [hemoglobin (Hg): 10.4 (9.37–12.6) vs. 12.1 (11–13.2) g/dL; *p* = 0.041]. *Conclusions*: Patients under chronic beta-blocker therapy undergoing non-cardiac surgery have a higher risk of perioperative bradycardia and hypotension. Continuous Holter monitoring proved effective in detecting transient arrhythmic events, emphasizing the need for careful perioperative surveillance of these patients, especially the elderly, in order to prevent cardiovascular complications These findings emphasize the necessity of tailored perioperative beta-blocker strategies and support further large-scale investigations to optimize risk stratification and management protocols.

## 1. Introduction

Beta-blocker treatment has been a milestone for many cardiac conditions and has been shown to be very effective in reducing heart rate, cardiac oxygen consumption, and contractility [1]. For these reasons, they help to prevent arrhythmias and have a cardioprotective role in general [2,3]. Many clinicians around the world recommend beta-blockers to treat heart conditions such as high blood pressure, ischemic heart disease, and heart failure and in rate control during atrial fibrillation because they lower the risk of adverse heart events [4]. Their main effect is beta-adrenergic receptor blockade, reducing sympathetic nervous system activation. This, in turn, lowers the stress and oxygen demand on the heart, subsequently reducing the heart rate [5]. Despite their extensively recorded advantages, the use of beta-blockers throughout the perioperative period in non-cardiac surgery has been a subject of significant contention and dispute. Although some studies have shown that beta-blocker medication usage correlates with decreased occurrences of perioperative myocardial infarction and reduced rates of postoperative atrial fibrillation [6,7], other research data suggest potential hazards. These concerns include a heightened probability of negative outcomes, including stroke, mortality, hypotension, and bradycardia [8,9]. The contradictory data have resulted in major variations in clinical practice and recommendations, with some healthcare professionals advocating for their use in certain patient categories, while others advise against their regular administration [10].

A key topic of disagreement is the administration of beta-blocker treatment prior to surgery in patients without proven cardiovascular risk factors or diseases. Recent recommendations indicate that the usual use of beta-blockers for prophylaxis in postoperative atrial fibrillation is not advised in patients without a preexisting cardiovascular illness [11]. This recommendation is based on findings that suggest that the potential harms of initiating therapy in previously untreated patients may outweigh the benefits. In contrast, for patients who are chronic users of beta-blockers, the current recommendations emphasize the importance of continuing therapy before, during, and after surgery to prevent adverse cardiovascular events. Studies have indicated that sustaining beta-blocker medication in chronic users during the perioperative phase is correlated with a decreased incidence of cardiovascular complications and lower death rates. Specifically, adherence to beta-blocker treatment on the day of and the day following surgery has been associated with fewer cardiovascular events and reduced 90-day mortality [12]. Conversely, the withdrawal of beta-blockers in the perioperative phase has been demonstrated to dramatically increase the risk of unfavorable cardiovascular events, including increased postoperative and one-year death rates. In addition, the discontinuation of beta-blockers has been linked with increased cardiovascular morbidity, including myocardial ischemia and arrhythmias [13]. On the other hand, some data have shown that beta-blocker discontinuation during the perioperative period may be connected to reduced demands for vasopressor support during postoperative care and shorter hospitalizations in the critical care unit [13,14]. This discovery raises issues concerning the ideal balance between the hazards and benefits of beta-blocker medication in the perioperative period, such as in non-cardiac and cardiac procedures. While beta-blockers offer considerable cardiovascular protection, their potential to contribute to perioperative hemodynamic instability necessitates cautious patient selection and personalized treatment techniques.

Despite the vast number of studies undertaken on this issue, there remains a lack of agreement about the relevance of beta-blockers in the perioperative period for non-cardiac procedures. To address this gap, our research aimed to examine the association between chronic beta-blocker therapy and perioperative cardiac events by utilizing data from 24 h continuous Holter monitoring, focusing on various complications, such as severe bradycardia, hypotension, atrial fibrillation with a rapid ventricular rate, and sustained ventricular tachycardia. The objective of the research was to analyze perioperative cardiac events in patients on chronic beta-blocker treatment utilizing continuous Holter monitoring. Holter monitoring allows the real-time, continuous detection of transient cardiac arrhythmias, which may go unnoticed in standard perioperative assessments. By offering detailed insights into cardiac rhythm stability, this research is intended to add to the continuing discussion on the best use of beta-blockers during surgeries. The novelty of this research resides in the use of continuous perioperative cardiac monitoring to examine the influence of chronic beta-blocker medication on perioperative cardiac events in patients having elective or emergency non-cardiac procedures. This approach offers more thorough knowledge of how beta-blockers alter cardiac rhythm stability and hemodynamic parameters throughout the perioperative period. The results from this research have the potential to impact therapeutic recommendations and offer vital data to guide patient management regimens, ensuring that the advantages of beta-blocker medication are maximized while limiting possible hazards.

## 2. Materials and Methods

### 2.1. Study Group Characteristics

A prospective observational study was conducted on 100 consecutive patients (63% male, 37% female; mean age 53.7 years) undergoing elective or emergency non-cardiac surgery at a Romanian tertiary care hospital. Inclusion criteria included age >18 years, the capacity to comply with perioperative monitoring, and the provision of informed consent. Surgical conditions encompassed acute cholecystitis, appendicitis, intestinal obstructions (volvulus, adhesions, intussusception), abdominal wall defects (inguinal, umbilical, and incisional hernias), splenic ruptures, gastric ulcers, gastrointestinal malignancies, and benign gynecological conditions. Patients were excluded if they were under 18 years old, if they refused or were unable to provide informed consent, or if continuous Holter monitoring could not be performed due to technical or logistical reasons. Additional exclusion criteria included acute coronary syndrome within the last 90 days, advanced heart failure (New York Heart Association Class III or IV), severe valvular diseases such as severe aortic stenosis or mitral regurgitation, chronic kidney disease with an estimated glomerular filtration rate (eGFR) ≤ 30 mL/min/1.73 m^2^, and an unstable preoperative hemodynamic status necessitating vasopressor support.

### 2.2. Ethical Aspects

This study was conducted in accordance with the Romanian Constitutional Law 206/2004 (updated 2021), the Declaration of Helsinki, and European Union regulations on professional ethics. Ethical approval was granted by the Ethics Committee of Scientific Research at Carol Davila University of Medicine and Pharmacy, Bucharest (37582/17 December 2024). Written informed consent was obtained from all participants prior to enrolment. The consent form explicitly guaranteed patient anonymity, confidentiality, and the right to withdraw without affecting medical care. Data collection and handling complied with institutional guidelines for personal data protection.

### 2.3. Study Design

This was a unicentric, prospective, non-interventional study. Data were collected from medical records, anesthesiology protocols, surgical reports, and 24 h Holter monitoring (initiated 1 h preoperatively and continuing for 24 h postoperatively). Hemodynamic parameters, including heart rate and blood pressure, were measured and recorded at standardized intervals: at hospital admission prior to anesthesia, intraoperatively every 15 min, immediately postoperatively, and at discharge from the recovery unit. Additional data collected included oxygen saturation, arrhythmia episodes (detected by Holter), the need for pharmacologic or electric cardioversion, vasopressor or inotropic agent administration, and any episodes of coronary ischemic syndrome, as indicated by ECG changes or troponin elevation. Holter ECG recordings were manually reviewed and interpreted by two independent cardiologists, each with extensive experience. This dual-review process was implemented to ensure accuracy in arrhythmia detection and to minimize the impact of artifacts on the final data interpretation. 

### 2.4. Sample Size

Sample size calculation was performed using the G*Power software, version 3.1 (Heinrich-Heine-Universität Düsseldorf, Düsseldorf, Germany). Based on the previous literature, we assumed a perioperative cardiac complication rate of approximately 40% in chronic beta-blocker users, compared to 10% in non-users. A chi-squared test was selected for the comparison of two independent proportions, with a two-sided significance level (α) of 0.05, power (1-β) of 0.80, and an effect size (w) of 0.35, corresponding to a moderate to large effect. The G*Power analysis indicated a minimum required total sample size of 64 patients. A minimum of 27 patients was required in the treated group. To allow for up to 10% attrition and to improve the precision in our models, we enrolled 30 chronic β-blocker users and 70 controls. Our study included 100 patients, exceeding the calculated requirement and thus ensuring adequate statistical power to detect clinically significant differences and minimizing the risk of type II errors.

### 2.5. Statistical Analysis

All statistical analyses were conducted using the Statistical Package for the Social Sciences (SPSS, version 26.0, IBM Corp., Armonk, NY, USA). Descriptive statistics were used wherever necessary to summarize patient characteristics such as age and gender, which were of a normal distribution, while the remaining continuous variables were of a non-normal distribution according to the Shapiro–Wilk test. The study utilized a chi-squared test to analyze variables, specifically assessing the association between beta-blocker use and perioperative complications. Pearson’s chi-squared values, degrees of freedom, and *p*-values were reported to determine statistical significance. The likelihood ratio test and linear-by-linear association test were also applied to validate the relationships between categorical variables. A binary logistic regression analysis was performed to evaluate the impact of specific cardiac conditions (e.g., atrial fibrillation, left bundle branch block) on beta-blocker use. The model’s predictive accuracy was assessed using the Omnibus Test of Model Coefficients, and the model fit was quantified using Nagelkerke’s R-squared. A *p*-value < 0.05 was considered statistically significant, and all results were reported with 95% confidence intervals. Furthermore, to assess for potential confounding by baseline age and smoking status, we applied (1) a logistic regression model for the composite ECG endpoint (≥1 new arrhythmia/conduction event), including age (per decade) and current smoking, and (2) a linear regression model for the minimum perioperative systolic blood pressure, with the same covariates. These analyses ensured a robust assessment of beta-blockers’ effects in the perioperative period.

## 3. Results

This study included 100 patients (63% male, 37% female), with a mean ± SD age of 50.2 ± 17.1 years in G1 and 58.5 ± 16.2 years in G2, indicating that patients in G2 were older. Furthermore, G1 had a larger proportion of smokers compared to G2. Surgical conditions were diverse, with cholecystitis being the most common (23 patients), followed by appendicitis (10), inguinal hernia (9), and colon cancer (8). Cardiovascular diseases included hypertension (47 patients) and atrial fibrillation (25 patients). Beta-blockers were prescribed to 30 patients in the G2 group, primarily for atrial fibrillation or hypertension, and the most commonly used beta-blockers were metoprolol and bisoprolol. The mean height of the patients included in the study was 170 cm, and the mean weight was 83.6 kg. All patients included in the dataset were of Romanian ethnicity. The demographic characteristics are presented in Table 1. Regarding comorbidities, hypertension was present in 51% of patients (*n* = 51), with a higher prevalence in the beta-blocker group. Dyslipidemia was noted in various forms: 41% of all patients had no dyslipidemia, 33% had controlled dyslipidemia, and 26% had uncontrolled dyslipidemia. Diabetes mellitus was diagnosed in 24% of the overall cohort, equally distributed across both groups: 12 patients with diabetes were present in the beta-blocker group and 12 in the non-beta-blocker group. In terms of surgical characteristics, 59% of the patients underwent emergency surgery and 41% had elective procedures. General anesthesia was used in 69% of cases, while 31% received spinal anesthesia. The types of surgical pathologies for which patients were admitted to the hospital are presented in Table 2. Furthermore, the American Society of Anesthesiologists (ASA) score is a physical status classification system; it is a fundamental tool in perioperative medicine, providing healthcare professionals with a standardized method to assess and categorize patients’ physiological status before surgery. The characteristics, along with the ASA scores, of the patients are summarized in Table 3.

### 3.1. Beta-Blockers Indication and Complications of Beta-Blocker Treatment

**Figure 1:**
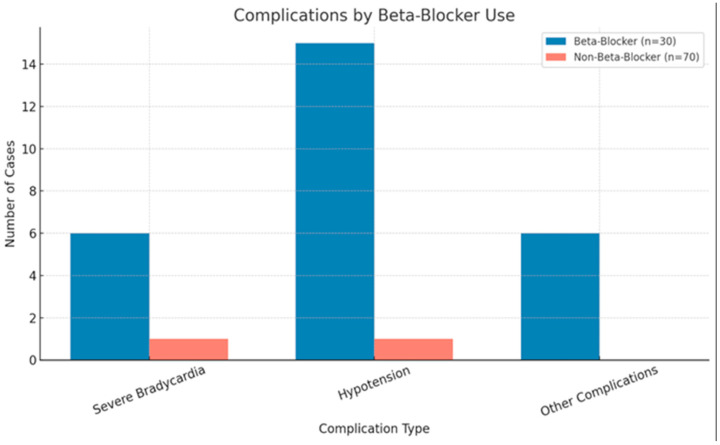
Perioperative cardiac events between G1 and G2 groups.

The chi-squared test was utilized to analyze the two categorical variables, i.e., beta-blocker indication—where 0 indicated no beta-blockers, 1 indicated ventricular extrasystoles, 2 indicated atrial fibrillation with a rapid ventricular rate (RVR), 3 indicated HTA, 4 indicated myocardial ischemia, 5 indicated no justification for beta-blocker use—and the consequences of betablocker treatment—where 0 indicated no beta-blocker use, 1 indicated no cardiac diseases, 2 indicated severe bradycardia, 3 indicated hypotension, and 4 indicated an AV block. The test indicated a substantial correlation between the need for beta-blockers and the observed complications. A total of 100 cases were evaluated, with no missing data. The majority of cases (70 patients out of 100 patients) fell within the “no beta-blocker use” category, with complications being rare in this group. However, when beta-blockers were necessary, different complications occurred, such as severe bradycardia (six instances) and hypotension (15 cases) (Figure 1). The chi-squared test demonstrated a substantial link, as the Pearson chi-squared value was 98.485, with 12 degrees of freedom and a *p*-value < 0.001, demonstrating a statistically significant relationship between beta-blocker use and complications, with a risk ratio (RR) of 2.96 (95% CI 1.84–4.75) (Table 4, Table 5 and Table 6). Similarly, the likelihood ratio and linear-by-linear relationship also revealed significant findings (*p* < 0.001). The requirement for beta-blockers correlated statistically significantly with more serious consequences, such as severe bradycardia and hypotension. These results underline the significance of constantly monitoring patients on beta-blockers for possible harmful effects. We also performed a logistic regression of any ECG abnormality in relation to age (per 10-year increase) and smoking status, which yielded a trend toward lower odds of new ECG findings with increasing age (OR 0.72 per decade, *p* = 0.07) and a non-significant increase with smoking (OR 1.67, *p* = 0.14). These findings are shown in Table 7. A linear regression of the minimum perioperative systolic BP on age (in years) and smoking status indicated that older patients tended to have a slightly lower nadir BP (≈0.24 mm Hg decrease per year of age, *p* = 0.06), while smoking status had no appreciable effect on the lowest systolic BP (*p* = 0.79) (Table 7). The linear regression of the lowest perioperative SBP on age, smoking, and anesthesia revealed that patients under spinal anesthesia had a significantly higher nadir SBP by ~12 mm Hg compared to those under general anesthesia (*p* = 0.009), after adjusting for age and smoking (Table 8).

When we ran the subgroup analysis by surgical urgency, the association between perioperative beta-blocker use and postoperative events remained strong in both elective and urgent procedures. In the elective cases, among the 31 patients not on beta-blockers, only one experienced a severe complication, compared with 4 out of 11 of those on beta-blockers. Meanwhile, among the 39 patients not on beta-blockers in the no beta-blocker group, one complication was observed, versus 13 out of 19 in the beta-blocker group. There was no evidence that urgency, whether urgent or elective, materially confounds the beta-blocker association. The findings are shown in Table 9.

### 3.2. Beta-Blockers and Surgery Duration

The analysis of beta-blocker use in relation to the surgery duration was adjusted for the surgical pathology type to account for potential confounding by procedural complexity. After this adjustment, no statistically significant association was found between beta-blocker use and the surgery duration (Pearson chi-squared = 9.351, df = 6, *p* = 0.155). This finding was consistent across alternative statistical approaches (likelihood ratio: 8.983, *p* = 0.175; linear-by-linear association: 3.608, *p* = 0.058). Table 3 presents the distribution of surgery durations across both groups, showing similar patterns regardless of beta-blocker use. Importantly, the surgical pathology type was the primary determinant of the operative time (*p* < 0.001), while beta-blocker administration showed no independent effect. The findings are presented in Table 10 and Table 11.

### 3.3. Beta-Blocker Indication and Sustained Ventricular Tachycardia (sTV)

We next examined the relationship between beta-blocker indication and the occurrence of sustained ventricular tachycardia (sTV). Out of 100 cases, 70 patients did not require beta-blockers, with 68 showing no sTV and only two experiencing sTV. Among those with conditions requiring beta-blockers (such as atrial fibrillation, hypertension, and myocardial ischemia prevention), the number of sTV cases was minimal (only 3 out of 100 cases). The chi-squared test results indicated no statistically significant association between beta-blocker indication and sustained ventricular tachycardia. The Pearson chi-squared value of 1.325 (df = 4, *p* = 0.857) suggests that the differences observed were likely due to chance. Similarly, the likelihood ratio (1.580, *p* = 0.812) and linear-by-linear association (0.006, *p* = 0.937) further confirmed the absence of a significant relationship (Table 12). The results suggest that the indication of beta-blocker use does not significantly impact the occurrence of sustained ventricular tachycardia (sTV). While beta-blockers are often prescribed for arrhythmia control, their indication in either preventing or triggering sTV appears statistically non-significant in this dataset.

### 3.4. Effects of Beta-Blockers on Specific Conditions

The binary logistic regression analysis examined conditions potentially associated with beta-blocker use. Beta-blockers are typically indicated for these conditions; however, patients receiving beta-blockers may still experience them. These conditions included an atrioventricular block, a left bundle branch block, ventricular extrasystoles, and sustained ventricular tachycardia (Table 13). The model was statistically significant (χ^2^ = 22.417, df = 4, *p* < 0.001), demonstrating that the predictors significantly contributed to the likelihood of beta-blocker indication. The Nagelkerke R-squared value of 0.341 suggested that the model explained 34.1% of the variance in beta-blocker prescription. The classification improved from 70% accuracy in the baseline model to 81% in the full model, with 92.9% accuracy in predicting non-users of beta-blockers and 53.3% accuracy in predicting those who required them. Among the predictors, a left bundle branch block showed borderline statistical significance (*p* = 0.064, Exp (B) = 21.028), and ventricular extrasystoles approached significance (*p* = 0.057, Exp (B) = 2.796). However, sustained ventricular tachycardia (*p* = 0.408) and an atrioventricular block (*p* = 0.484) were not significant predictors.

#### New-Onset Paroxysmal Atrial Fibrillation

Ten patients developed paroxysmal postoperative AF, which was related to longer surgery durations (165 (150–180) vs. 120 (90–150) minutes (*p* = 0.002)) and postoperative anemia [hemoglobin (Hg) 10.4 (9.37–12.6) vs. 12.1 (11–13.2) g/dL, (*p* = 0.041)].

## 4. Discussion

Our study found a significant association between the use of beta-blockers and the occurrence of complications such as severe bradycardia, hypotension, and atrial fibrillation with a rapid ventricular rate (RVR). The chi-squared analysis indicated a statistically significant relationship (*p* < 0.001), highlighting that patients requiring beta-blockers were more likely to experience cardiovascular instability, which leads to acute perioperative hemodynamic disturbances or arrhythmias that compromise adequate blood flow, tissue perfusion, or cardiac rhythm stability. However, no significant association was found between beta-blocker use and the surgery duration (*p* = 0.155) or between beta-blocker indication and sustained ventricular tachycardia (*p* = 0.857). The binary logistic regression identified atrial fibrillation as the most significant predictor of beta-blocker indication (*p* < 0.001), while other complications had weaker correlations. These findings underscore the importance of tailored perioperative surveillance when administering beta-blockers perioperatively to mitigate potential risks.

Our findings align with previous studies that support the continuation of beta-blocker therapy in chronic users during the perioperative period to reduce cardiovascular complications. Kwon et al., in their 2012 research study, determined that maintaining beta-blocker therapy during and after surgery was linked to a lower incidence of cardiac complications and a decrease in 90-day mortality. Their findings highlighted the importance of continuing beta-blockers to mitigate the risk of perioperative myocardial ischemia and adverse outcomes. The study observed that patients who discontinued beta-blockers had nearly twice the likelihood of experiencing severe cardiac events, with even greater risks for those with an elevated baseline cardiovascular risk. These results emphasize the protective benefits of sustained beta-blocker use in surgical patients. A recent large-scale multicenter cohort study involving over 10,000 patients found that preoperative beta-blocker use was not associated with a reduction in perioperative myocardial infarctions or major adverse cardiac events or with increased mortality at 30 or 365 days [15]. These findings align with our results, which showed no significant benefit of beta-blockers in preventing ventricular tachyarrhythmias or influencing the duration of surgery. However, this study focused on beta-blockers in elective and emergency non-cardiac surgeries [12]. Similarly, Blessberger et al. reported, in a Cochrane review, that beta-blockers significantly reduced the incidence of perioperative atrial fibrillation and myocardial infarction; however, the authors also highlighted uncertainties and potential risks, such as increased bradycardia and hypotension [10]. In another research study by Blessberger et al., they investigated patients undergoing cardiac surgeries. By analyzing data from 63 studies with 7768 participants, they reached the conclusion that there was no indication of a difference in myocardial infarction, cerebrovascular events, bradycardia, hypotension, or early all-cause mortality. Conversely, the authors concluded that beta-blockers may reduce ventricular arrhythmias and atrial fibrillation. Additionally, Oesterle et al. conducted a meta-analysis demonstrating that beta-blockers can effectively prevent postoperative atrial fibrillation in non-cardiac surgeries. Their meta-analysis of 21 studies, with 11,608 patients, found that beta-blockers significantly reduced the incidence of postoperative atrial fibrillation (risk ratio [RR] 0.32; 95% confidence interval [CI] 0.11 to 0.87) compared to placebos or active controls, which aligns with Blessberger’s findings. This also aligns with our findings, as our binary logistic regression revealed that atrial fibrillation was the strongest predictor of beta-blocker indication (*p* < 0.001). However, Blessberger also noted that beta-blockers were associated with an increased risk of mortality and bradycardia, primarily driven by the POISE trial [16]. These studies reinforce our findings, highlighting the protective benefits of beta-blockers in patients undergoing non-cardiac surgeries, but there is still conflicting evidence among existing research, which must be further explored. In our study, among the ten patients who developed postoperative paroxysmal AF, only one was receiving chronic beta-blocker therapy, suggesting that the majority of AF cases were likely unrelated to beta-blocker exposure. Our data showed a significant association between new-onset AF and both a longer surgical duration and lower postoperative hemoglobin levels, pointing toward intraoperative physiological stress and anemia as more probable triggers. Although specific data on intraoperative blood loss or fluid balance were not collected, these findings emphasize the complex, multifactorial etiology of postoperative AF and the importance of maintaining both hemodynamic and hematologic stability in the perioperative period, regardless of beta-blocker use.

In addition, our findings contrast reports indicating that beta-blockers may increase perioperative risks. Wijeysundera et al. conducted a systematic review and meta-analysis to evaluate the impact of perioperative beta-blockers on patients undergoing non-cardiac surgery [9]. Their findings revealed that, while beta-blockers were associated with a reduction in non-fatal myocardial infarctions (RR: 0.69; 95% CI: 0.58 to 0.82), they significantly increased the risk of non-fatal stroke (RR: 1.76; 95% CI: 1.07 to 2.91) and all-cause mortality (RR: 1.30; 95% CI: 1.03 to 1.64) when excluding the controversial DECREASE trials [9]. The review further indicated that beta-blockers also raised the likelihood of adverse effects such as hypotension and bradycardia. Even after removing data from the POISE-1 trial, which had a significant influence on the results, the trend toward increased mortality persisted. A recent meta-analysis by Herrera Hernández et al. [17] reported that perioperative beta-blockers significantly increased the risks of hypotension (RR 1.46) and bradycardia (RR 2.26) but showed no significant mortality reduction (RR 0.62, *p* = 0.05) across 1.3 million patients. Notably, their study highlighted a heightened stroke risk (RR 1.42, *p* = 0.03), reinforcing the need for cautious patient selection [17]. These findings suggest that, while beta-blockers may lower the incidence of perioperative myocardial infarction, this potential benefit is counterbalanced by the heightened risks of stroke, mortality, and hemodynamic instability [9]. Friedell et al. examined the impact of perioperative beta-blockade in non-cardiac surgery and found that, while these medications provided benefits for patients with multiple cardiac risk factors, they posed significant risks for those without preexisting cardiovascular diseases [8]. Their analysis revealed that beta-blockers could lead to severe hemodynamic instability, including profound hypotension and bradycardia, which contributed to increased mortality in low-risk patients. Similarly, Bhave et al. updated the perioperative cardiovascular management guidelines, reinforcing concerns about the initiation of beta-blockers in patients without prior cardiovascular diseases [11]. The guidelines advised against starting beta-blockers perioperatively in beta-blocker-naïve patients due to evidence linking their use to higher rates of stroke, mortality, and adverse hemodynamic effects [8,11]. These findings underscore the importance of carefully selecting patients for beta-blocker therapy, prioritizing continuation in those already on treatment while avoiding indiscriminate initiation in those without a clear cardiovascular indication. These contrasting findings highlight the ongoing debate regarding the perioperative use of beta-blockers. The data and research highlight the dual nature of perioperative beta-blocker use. For patients under chronic beta-blocker therapy, especially those with arrhythmic or ischemic heart disease, continuation appears beneficial, aligning with large-scale reviews showing decreased arrhythmic events and improved survival [10,12]. Conversely, initiating beta-blockers in low-risk patients solely to forestall perioperative atrial fibrillation may lead to excessive bradycardia, hypotension, and potentially worse outcomes [8,9,10]. This emphasizes the need for a tailored approach to their administration and further research to assess the safety profiles of the medications perioperatively and postoperatively.

Another important aspect is the role of both acute and chronic inflammation in promoting arrhythmogenesis and perfusion disorders [18,19,20]. An imbalance in pro- and anti-inflammatory factors is directly responsible for the occurrence of electrolyte, acid–base, and hormonal imbalances. These metabolic imbalances, which generate systemic inflammation, also have secondary cardiovascular effects of variable severity, from silent, apparently asymptomatic ischemia to myocardial infarction or severe cardiac arrhythmias [21,22,23]. There is evidence that certain beta-blockers can be used in acute inflammatory states, with the effect of modulating neutrophil hyperactivation in the acute phase [24]. This supports the claim that beta-blockers have a cardioprotective effect. However, this evidence is inconclusive and heterogeneous.

Our findings support the use of continuous Holter monitoring as a sensitive tool for the detection of transient arrhythmic events, including paroxysmal atrial fibrillation, bradyarrhythmias, and occasional wide QRS complex tachycardias. While our protocol utilized standard single-lead Holter recording, we acknowledge the clinical value of 12-lead Holter ECG systems in providing a more precise differential diagnosis between supraventricular and ventricular tachycardias. As highlighted in the recent literature, including descriptions of advanced signal analysis and vector orientation assessment (ECG axis), the distinction between these arrhythmias is crucial, particularly in the perioperative setting, where rapid decisions impact management strategies [25,26].

Beyond rhythm classification, beta-blockers exert antiarrhythmic effects through multiple mechanisms. At the molecular level, they antagonize β1-adrenergic receptors in cardiac myocytes, attenuating the cAMP-mediated activation of protein kinase A, which in turn reduces the phosphorylation of calcium channels and ryanodine receptors. This leads to decreased intracellular calcium overload, a known substrate for delayed afterdepolarizations and triggered arrhythmias. At the cellular level, beta-blockers stabilize the membrane potential and prolong the action potential duration in atrial and ventricular tissue, limiting re-entry circuits. Tissue-level effects include the modulation of sympathetic tone and improvements in the myocardial oxygen supply–demand balance, further reducing the arrhythmogenic potential. These mechanisms justify their use not only in rate control but also in arrhythmia prevention across a range of clinical settings [1,2,3]

### 4.1. Limitations of the Study

First, this was a unicentric research study undertaken at a single institution, limiting the generalizability of the results. The study enrolled 100 consecutive patients, of whom 30 were on chronic beta-blocker therapy. The sample size was determined based on an expected difference in the perioperative cardiovascular complication rates between groups, as previously detailed. While the chosen cohort provided sufficient power to detect moderate effect sizes in the occurrence of bradycardia and hypotension, we acknowledge that the study may have been underpowered to capture rare but clinically important events, such as perioperative myocardial infarctions, cardiac arrest, or stroke. Moreover, the single-center design and the regional characteristics of the patient population may limit the external validity of the findings. These aspects underscore the need for future multicenter trials with larger sample sizes and diverse populations to validate and expand upon our results.

Another limitation of our study is the heterogeneity of beta-blocker therapy among the patients in the chronic treatment group. Although most received cardio-selective agents such as metoprolol or bisoprolol, the doses were not standardized. This reflects real-world practice but may have influenced the incidence of bradycardia and hypotension, as different beta-blockers have distinct pharmacologic profiles. Future studies should account for the beta-blocker type and dosage when evaluating the perioperative risk.

The observational approach does not allow for the establishment of causality, since unmeasured confounders may have impacted the outcomes. Holter monitoring was restricted to 24 h postoperatively, possibly missing late-onset cardiac incidents. Changes in surgical procedures, anesthetic techniques, and patient comorbidities may also have impacted the findings. Furthermore, our analysis controlled for the surgical type, but residual confounding may have existed due to variability in procedural complexity within categories. Another limitation of our study is the inclusion of both elective and emergency surgical procedures, which may have introduced heterogeneity in perioperative management and potentially confounded the relationship between perioperative beta-blocker use and postoperative cardiac events. Although the urgency of surgery was recorded, a formal subgroup analysis was not performed. However, exploratory analyses did not suggest significant differences in the distribution of beta-blocker use or cardiac outcomes between elective and emergency cases. Nevertheless, we acknowledge that this heterogeneity may impact the generalizability and internal validity of our findings. Another limitation of our study is the lack of detailed data regarding intraoperative fluid shifts and blood loss. These factors can impact hemodynamic stability and may influence the occurrence of perioperative arrhythmias or hypotension.

Another limitation of our study is the interval-based nature of blood pressure (BP) monitoring. While cardiac electrical activity was recorded continuously using a Holter device, BP measurements were performed at standardized 15 min intervals intraoperatively. Although this protocol aligns with institutional practice, it may have failed to capture transient episodes of hypotension, particularly during anesthesia induction or emergence—phases known to carry a higher risk of hemodynamic instability. Continuous non-invasive BP monitoring could provide more granular data and should be considered in future studies assessing perioperative cardiovascular dynamics, especially in patients under beta-blocker therapy [27]

Lastly, given that our research did not link beta-blockers to sustained ventricular tachycardia, it implies that the short-term arrhythmogenic risk may be limited for this specific rhythm abnormality. Nonetheless, only prolonged monitoring (beyond our 24 h timeframe) can clarify whether late arrhythmic episodes are being overlooked. Moreover, future multicentric trials and randomized controlled trials with larger populations and prolonged monitoring periods are required to corroborate our results.

Although several perioperative cardiac events, such as severe bradycardia, hypotension, and arrhythmias, were recorded—particularly among patients on chronic beta-blocker therapy—these events were promptly recognized and managed, without leading to major adverse outcomes, such as myocardial infarction, stroke, or in-hospital death. No cases required prolonged ICU admission or resulted in an increased overall length of hospitalization. While quality of life and long-term prognosis were not formally assessed, all patients were discharged in a stable condition. These findings suggest that, although beta-blocker-associated hemodynamic events are clinically relevant, their short-term consequences may be mitigated through close monitoring and timely intervention.

### 4.2. Future Implications

Our study offers significant insights into the perioperative care of patients on persistent beta-blocker therapy; nevertheless, more research is required to enhance therapeutic guidelines. The results underscore the necessity of personalized risk evaluation when prescribing beta-blockers in the perioperative context. Subsequent research should investigate larger, multicentric populations to improve the generalizability of the findings and validate the identified interaction. Furthermore, prolonged Holter monitoring beyond 24 h may facilitate the detection of delayed cardiac events that were not seen in our investigation. Future studies can consider prolonged monitoring; however, it might prove to be costlier [2]. Considering the substantial correlation between beta-blocker administration and complications including severe bradycardia and hypotension, further research should focus on refining perioperative beta-blocker protocols to reduce hemodynamic instability. Exploring different dosage regimens, patient selection criteria, and intraoperative monitoring approaches may enhance the safety and efficacy. Our data also prompt inquiries regarding the optimal timing of beta-blocker administration. Future studies may evaluate whether changes in preoperative dose or intraoperative modifications mitigate adverse events while maintaining cardiovascular protection. Furthermore, research contrasting beta-blockers with other cardioprotective medications may elucidate the optimal strategy for high-risk surgical patients. Our results advance the discourse on perioperative beta-blocker use and provide a basis for evidence-based recommendations that enhance patient outcomes while mitigating possible hazards.

Future research should aim to validate our findings in larger, multicenter cohorts and through randomized controlled trials comparing the continuation versus discontinuation of beta-blockers in the perioperative setting. Stratification by beta-blocker type and dosage may help to clarify differential risk profiles, while extended Holter monitoring could uncover late-onset arrhythmic events beyond the initial 24 h. Additionally, preoperative Holter assessments in selected high-risk patients may offer a useful tool for individualized risk stratification. These steps could contribute to a more nuanced and evidence-based approach to perioperative beta-blocker management.

## 5. Conclusions

This prospective Holter-based analysis demonstrates that chronic beta-blocker therapy in patients undergoing non-cardiac surgery is significantly associated with an increased incidence of perioperative bradycardia and hypotension. Importantly, no correlation was observed between beta-blocker use and the occurrence of ventricular tachyarrhythmias in the perioperative period. These findings underscore the dual nature of beta-blockers in the surgical context—while they offer potential cardioprotective benefits, they also carry measurable hemodynamic risks. Therefore, optimal perioperative use requires careful patient selection, individualized risk–benefit assessment, and rigorous intraoperative and postoperative monitoring. The use of continuous Holter monitoring proved valuable in capturing transient but clinically relevant cardiac events, highlighting its utility in perioperative surveillance. Further large-scale, multicentric studies with extended follow-up durations are warranted to refine clinical guidelines and enhance perioperative management protocols, ultimately promoting the safer and more effective use of beta-blockers in surgical populations.

## Figures and Tables

**Table 1:** Demographic characteristics of patients.

Group	*n*	Age (Mean ± SD)	BMI (Mean ± SD)	Smokers (%)
G2	30	58.5 ± 16.2	28.9 ± 3.6	36.7% (11/30)
G1	70	50.2 ± 17.1	28.8 ± 4.0	42.9% (30/70)
All Patients	100	52.9 ± 17.2	28.8 ± 3.9	41.0% (41/100)

**Table 2:** Distribution of surgical conditions.

Surgical Condition	G2 (*n*/%)	G1 (*n*/%)	Total (*n*)
Cholecystitis	8 (26.7%)	13 (18.6%)	21
Appendicitis	1 (3.3%)	8 (11.4%)	9
Inguinal Hernia	2 (6.7%)	10 (14.3%)	12
Colon Cancer	5 (16.7%)	5 (7.1%)	10
Umbilical Hernia	3 (10.0%)	7 (10.0%)	10
Perianal Fistula	2 (6.7%)	6 (8.6%)	8
Incarcerated Hernia	2 (6.7%)	3 (4.3%)	5
Postsurgical Adhesions	2 (6.7%)	5 (7.1%)	7
Gastric Ulcer	3 (10.0%)	1 (1.4%)	4
Endometrial Cancer	0 (0.0%)	2 (2.9%)	2
Gastric Cancer	0 (0.0%)	2 (2.9%)	2
Uterine Fibroma	0 (0.0%)	2 (2.9%)	2
Hemorrhoids	1 (3.3%)	2 (2.9%)	3
Splenectomy	0 (0.0%)	2 (2.9%)	2
Diverticulitis	1 (3.3%)	1 (1.4%)	2
Intestinal Volvulus	0 (0.0%)	1 (1.4%)	1
Total	30 (30.0%)	70 (70.0%)	100

**Table 3:** ASA scores, with the patient counts in the respective rows.

ASA Score	Count
1	25
1E	3
2	3
2E	33
3	12
3E	12
4	2
4E	10

**Table 4:** Complications of beta-blockers.

Complication	G2	G1
Severe Bradycardia	6 (20.0%)	1 (1.4%)
Hypotension	15 (50.0%)	1 (1.4%)
Other Complications *	6 (20.0%)	0 (0%)

* AV block, arrhythmias not categorized above; RVR: rapid ventricular rate; HTA = hypertension.

**Table 5:** Chi-squared tests.

Test	Value	Df	Asymptotic Significance (2-Sided)
Pearson Chi-Squared	98.485	12	<0.001
Likelihood Ratio	94.731	12	<0.001
Linear-by-Linear Association	54.947	1	<0.001
*n* of Valid Cases	100		

**Table 6:** Indications for beta-blockers.

Indication	G2
Permanent AF	14 (46.7%)
Hypertension (HTA)	11 (36.7%)
Extrasystolic arrhythmia	3 (10.0%)
Coronary ischemic syndrome	2 (6.7%)

**Table 7:** Effects of smoking and age on ECG events and perioperative hypotension.

Outcome	Variable	Estimate (OR or β)	95% CI	*p*-Value
Any new postoperative ECG finding	Age (per 10 years)	OR 0.72	0.61–1.02	0.07
	Smoker vs. non-smoker	OR 1.67	0.85–3.42	0.14
Minimum perioperative SBP (linear)	Age (per 1 year)	β –0.24 mm Hg	–0.50 to +0.01	0.06
	Smoker vs. non-smoker	β +1.14 mm Hg	–7.27 to +9.55	0.79

**Table 8:** Minimum intraoperative systolic BP (age, smoking, anesthesia type).

Variable	β (mm Hg)	95% CI	*p*-Value
Intercept	113.7	98.4–129.0	<0.001
Age (per 1 year)	−0.20	−0.44–+0.05	0.11
Smoker vs. non-smoker	+3.47	−4.86–+11.81	0.41
Spinal vs. general anesthesia	+12.17	+3.14–+21.19	0.009

**Table 9:** Complications in patients with and without beta-blockers, undergoing elective and urgent surgery.

Urgency	Group	*n*	Complication
Elective	G1	31	1
	G2	11	4
Urgent	G1	39	1
	G2	19	13

**Table 10:** Beta-blockers and surgery time in hours.

Beta-Blockers	1.0	1.5	2.0	2.5	3.0	4.0	4.5	Total
G1	8	11	22	14	12	2	1	70
G2	2	4	7	9	2	4	2	30
Total	10	15	29	23	14	6	3	100

**Table 11:** Chi-squared tests.

Test	Value	Df	Asymptotic Significance (2-Sided)
Pearson Chi-Squared	9.351	6	0.155
Likelihood Ratio	8.983	6	0.175
Linear-by-Linear Association	3.608	1	0.058
*n* of Valid Cases	100		

**Table 12:** Beta-blocker indication and sustained ventricular tachycardia.

Beta-Blocker Indication	Sustained Ventricular Tachycardia (No)	Sustained Ventricular Tachycardia (Yes)	Total
No Beta-Blockers	68	2	70
Ventricular Extrasystoles	3	0	3
Atrial Fibrillation with RVR	13	1	14
Hypertension (HTA)	11	0	11
Myocardial Ischemia Prevention	2	0	2
Total	97	3	100
Chi-Squared Tests			
Test	Value	Df	Asymptotic Significance (2-Sided)
Pearson Chi-Squared	1.325	4	0.857
Likelihood Ratio	1.580	4	0.812
Linear-by-Linear Association	0.006	1	0.937
*n* of Valid Cases	100		

**Table 13:** Beta-blockers and specific complications.

Variable	B	S.E.	Wald	Sig.	Exp (B)
Atrioventricular block	−0.842	1.205	0.489	0.484	0.431
Left bundle branch block	3.046	1.643	3.436	0.064	21.028
Ventricular extrasystoles	1.028	0.541	3.615	0.057	2.796
Sustained ventricular tachycardia	−1.353	1.634	0.686	0.408	0.258
Constant *	−2.077	0.445	21.818	<0.001	0.125

* Baseline log-odds of the outcome.

## Data Availability

All data generated or analyzed during this study are included in this article. Further enquiries can be directed to the corresponding author.
